# Visualization of segmental arterialization with arrival time parametric imaging using Sonazoid-enhanced ultrasonography in portal vein thrombosis: A case report

**DOI:** 10.3892/etm.2012.874

**Published:** 2012-12-21

**Authors:** NORITAKA WAKUI, RYUJI TAKAYAMA, YASUSHI MATSUKIYO, MIE SHINOHARA, SHUNSUKE KOBAYASHI, TAKENORI KANEKAWA, SHIGERU NAKANO, HIDENARI NAGAI, TAKAHIDE KUDO, KENICHI MARUYAMA, YASUKIYO SUMINO

**Affiliations:** 1 Division of Gastroenterology and Hepatology; Toho University Omori Medical Center, Ota-ku 143-8541, Tokyo, Japan; 2 Division of Clinical Functional Physiology, Toho University Omori Medical Center, Ota-ku 143-8541, Tokyo, Japan

**Keywords:** contrast-enhanced ultrasonography, Sonazoid, arrival time parametric imaging, portal thrombus, arterialization

## Abstract

A 55-year-old male was admitted in mid-April 2011 with a fever of >39°C and pain in the lower right abdomen. A medical examination revealed sepsis originating from colonic diverticulitis. Abdominal B-mode ultrasonography (US) performed on admission detected thrombi in the superior mesenteric vein and in the right branch of the hepatic portal vein. Arrival time parametric imaging (At-PI) using Sonazoid-enhanced US showed arterialization of the entire right lobe of the liver. The treatment for the sepsis and portal thrombi that had been started upon admission dissolved the thrombi by day 22, with the exception of one thrombus in the P8 branch of the portal vein. At-PI performed on the same day confirmed arterialization in segment 8, but portal vein dominance was restored elsewhere. When the blood inflow from the hepatic portal vein was reduced, the hepatic arterial blood flow was increased to compensate for the reduction in the total blood supply. The At-PI functions used in the Sonazoid-enhanced US were simple yet effective in visualizing the changes in the hepatic hemodynamics caused by the portal thrombus.

## Introduction

The liver receives a dual blood supply from the hepatic portal vein and the hepatic artery. The portal vein provides 70–80% of the supply, carrying nutrients and various other substances to the liver. The remaining 20–30% of the blood supply comes via the hepatic artery and mainly nourishes the biliary system (1). While the blood pressure of the arterial system exceeds 100 mmHg, the pressure of the portal vein is as low as 6–8 mmHg. Due to this pressure difference, when problems arise in the liver or other areas supplied by these vessels, the portal vein is usually the first vessel affected, therefore leading to a reduced blood flow. Such reduction in the total hepatic blood supply appears to activate a compensatory mechanism that increases the arterial blood flow (1–4).

Our group is studying hepatic hemodynamic changes in an effort to understand the pathology of liver disease.

In the present study, a case of acute portal vein thrombosis is reported that may reveal, in part, the mechanism that regulates the blood flow balance between the hepatic artery and the hepatic portal vein.

## Case report

### Patient history and presentation

**Figure 1.**
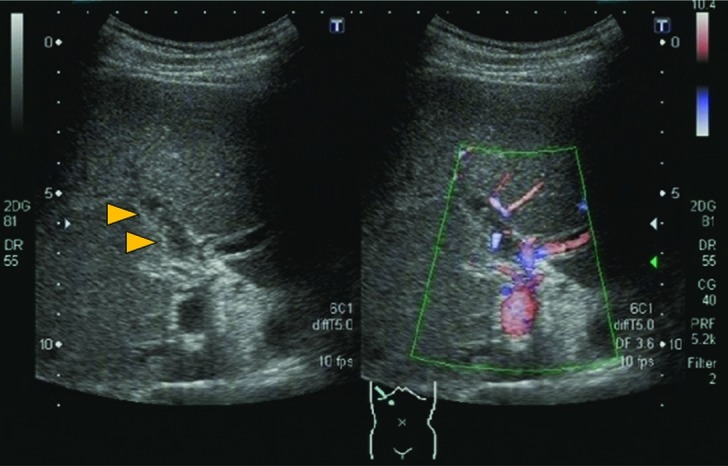
Abdominal ultrasonograpy (US) findings on admission. Left: B-mode US revealed a solid lesion with a mixed hyper- and hypoechoic pattern (arrowheads) in the right branch of the hepatic portal vein. Right: color Doppler US showed neither blood flow through the right branch nor the signs of portal vein dilatation.

**Figure 2.**
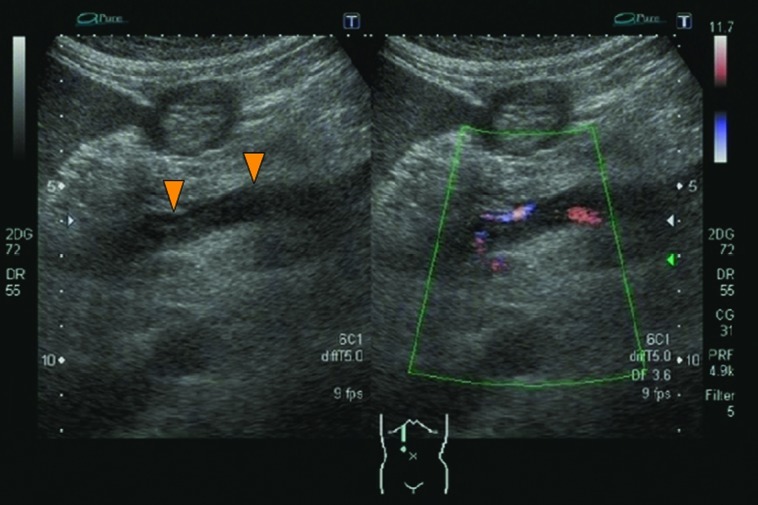
Abdominal ultrasonography (US) findings on admission. Left: B-mode US-detected thrombi (arrowheads) in the superior mesenteric vein. Right: color Doppler US showed neither blood flow through the superior mesenteric vein nor signs of dilatation.

**Figure 3.**
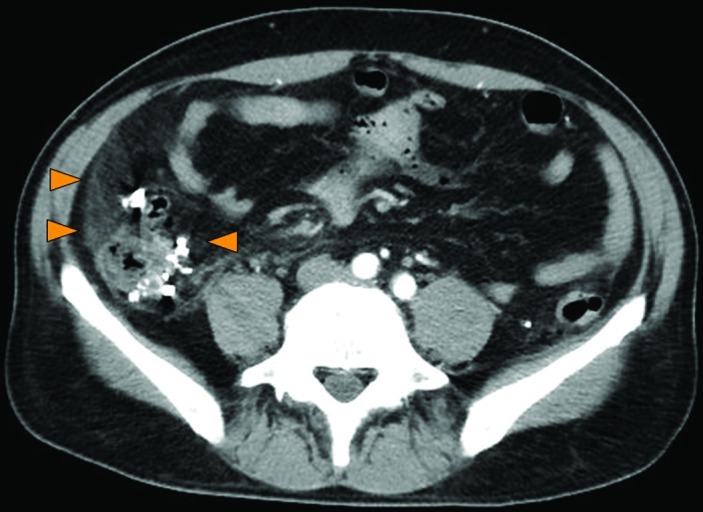
Abdominal computed tomography (CT) findings on admission. CT imaging revealed thickening of the ascending colon wall, numerous diverticula within and outside the colon wall and thickening of the surrounding fat layer (arrowheads).

This study was performed with approval of the Ethics Committee at Toho University Omori Medical Center (Tokyo, Japan). Written informed patient consent was obtained from the patient. A 55-year-old male developed a fever of ≥39°C and intermittent pain in the lower right abdomen in April 2011. The patient visited the Toho University Omori Medical Center 7 days subsequent to onset due to gradually intensifying pain and yellow eyes. The patient had no history of alcohol consumption or smoking, but was treated previously for cutaneous lupus erythematosus. His family history revealed nothing of note. At the initial visit, the patient was alert with a blood pressure of 140/76 mmHg, a heart rate of 100 bpm and a body temperature of 38.3°C. The palpebral conjunctiva showed no signs of anemia, but the bulbar conjunctiva revealed a yellowish discoloration. Pure heart sounds, clear breath sounds and a soft, flat abdomen were noted. Although the abdomen in the lower right quadrant was tender to touch, there was no muscular defense or rebound tenderness. The liver and spleen were not palpable and no edema was present in the lower extremities. The hematological findings showed 21.0 mg/dl C-reactive protein (CRP), a white blood cell (WBC) count of 16,900 cells/*μ*l, indicating a heightened inflammatory response, a reduced platelet (Plt) count of 37,000/*μ*l and high levels of fibrin degradation products (FDPs) and D-dimers. The levels of hepatic and biliary enzymes (Table I) were also increased. On the basis of these findings, the patient was admitted with suspected sepsis. Abdominal ultrasonography (US) performed on admission revealed a solid lesion with a mixed hyper- and hypoechoic pattern in the right branch of the hepatic portal vein with virtually no blood flow detected by color Doppler US. There was no portal vein expansion noted. From these findings, a diagnosis of right portal vein thrombosis was made (Fig. 1). The left branch of the portal vein was free of thrombi and had a normal blood flow. However, a thrombus was present in the superior mesenteric vein (Fig. 2), suggesting that the splenic vein and the inferior mesenteric vein were the source of the blood to the portal vein. There was also thickening of the ascending colon wall, numerous diverticula within and outside the colon wall and thickening of the surrounding fat layer, all of which led to a diagnosis of colonic diverticulitis (Fig. 3). These findings corresponded with the patient’s tender abdominal area.

### Clinical course and imaging findings subsequent to admission

Due to the presence of *Escherichia coli* in the blood culture subsequent to admission, the patient was diagnosed with sepsis caused by ascending colon diverticulitis. He was consequently started on 1.5 g/day of the antibiotic doripenem and 2 g/day of gabexate mesilate on hospital day 1, followed by 12,800 U/day thrombomodulin-α and 10,000 U/day heparin Na on day 2. Arrival time parametric imaging (At-PI) using Sonazoid-enhanced US was performed on hospital day 1 to assess the blood flow in the hepatic portal vein and the hepatic artery.

US imaging was performed using a Toshiba Aplio XG ultrasonographic device (model SSA-790A; Toshiba Medical Systems Co., Tochigi, Japan) with a 3.75-MHz convex array probe (PVT-375BT) at a mechanical index of 0.21. Images showing the liver parenchyma from the right intercostal space to segments 5–8 of the right hepatic lobe as well as the right kidney were captured for analysis. The focal depth was set at 8 cm to visualize the kidney. Subsequent to setting the imaging parameters, the recommended dose of Sonazoid (0.015 ml/kg; perfluorobutane; GE Healthcare, Oslo, Norway) was injected via the cubital vein. The sonographic data that were generated for a period of ∼40 sec following the Sonazoid infusion were stored as raw data in the system hardware.

**Figure 4.**
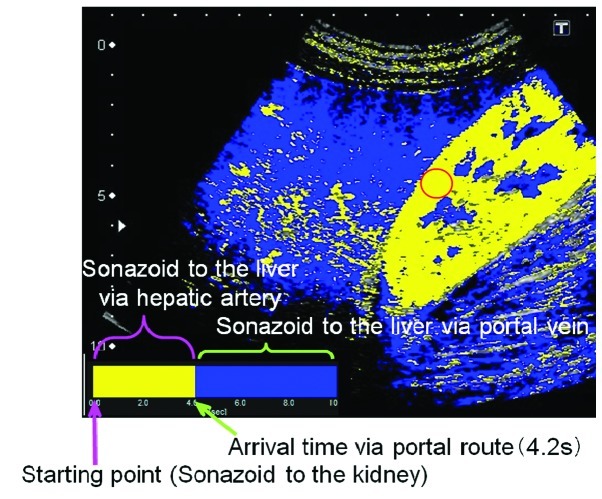
Arrival time parametric imaging (At-PI) differentiating the portal venous perfusion from the hepatic arterial perfusion. The renal enhancement starting time was set at 0. Yellow and blue colors represent pixels arriving prior to and subsequent to the visualization of the portal vein, respectively. Thus, perfusion from the hepatic artery and portal vein is shown in yellow and blue, respectively. As the left branch of portal vein was visualized 4.2 sec after renal enhancement in this study, 4.2 sec was used to create the color map of hepatic perfusion.

**Figure 5.**
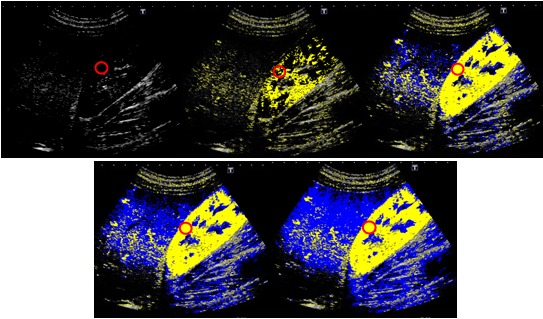
Procedure for arrival time parametric imaging (At-PI). Subsequent to setting the region of interest (ROI) in the kidney parenchyma, the movie was played back and the arrival times were calculated sequentially for each liver parenchymal pixel. The time point at which 80% of the ROI was enhanced by the contrast medium was defined as time 0. A color map was then automatically superimposed on a B-mode image. Pixels with an arrival time of 0–4.2 sec are displayed in yellow and those with an arrival time of >4.2 sec are displayed in blue.

At-PI of the stored data was performed using the software interfaced with the ultrasound system. By selecting the region of interest (ROI) within the kidney parenchyma, the point at which 80% of the ROI was contrasted was set as time 0 and the arrival time of individual pixels representing hepatic parenchymal enhancement were sequentially calculated. A color map was created and automatically superimposed on a B-mode image. In the present study, two colors were selected to differentiate the portal venous perfusion from the hepatic arterial perfusion. With time 0 starting at 80% of the renal enhancement, the pixels arriving prior to the visualization of the portal vein were displayed in yellow and those arriving subsequent to the visualization were displayed in blue. The 4.2 sec taken to visualize the left branch of the hepatic portal vein were used to discriminate the route of hepatic perfusion and thus color maps generated in the present study showed the period of hepatic arterial perfusion in yellow and that of portal venous perfusion in blue (Figs. 4 and 5).

**Figure 6.**
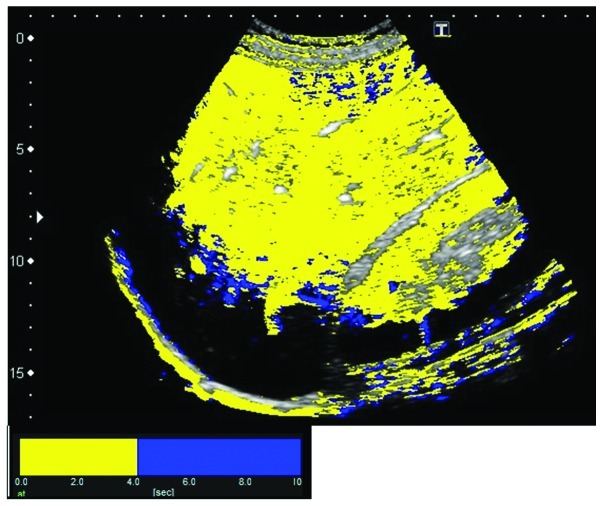
Arrival time parametric imaging (At-PI) findings on admission. Due to portal vein obstruction by a thrombus, the entire right lobe of the liver was highlighted in yellow, indicating that the hepatic parenchyma was perfused by the hepatic artery.

**Figure 7.**
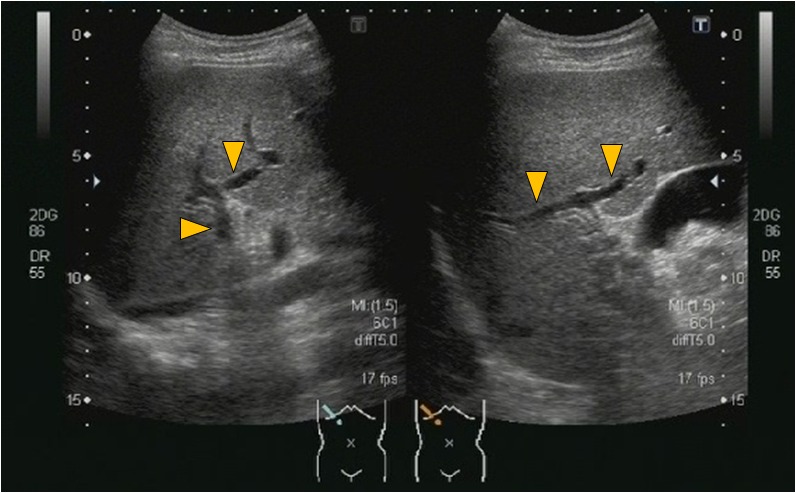
Abdominal ultrasonography (US) findings on hospital day 22. B mode US images show that the thrombus remaining in the right main branch of the portal vein had been mostly dissolved (arrowheads).

**Figure 8.**
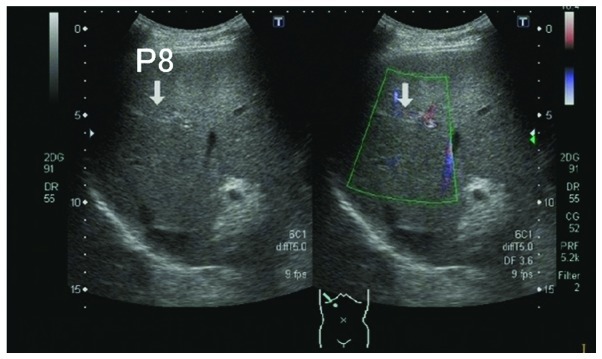
Abdominal ultrasonography (US) findings on hospital day 22 show the remaining thrombus in the P8 branch of the portal vein (arrow).

**Figure 9.**
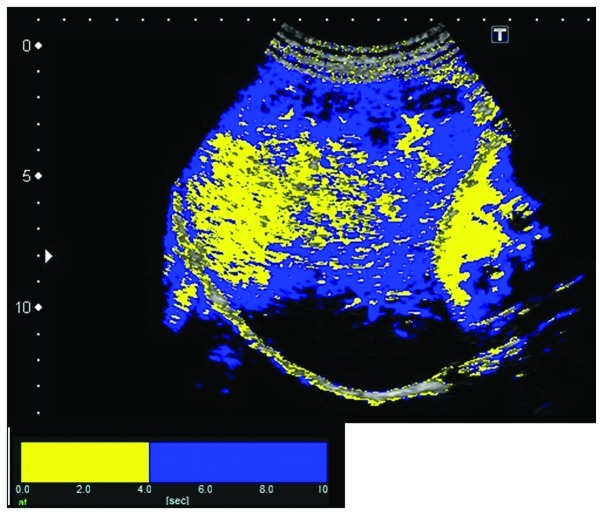
Arrival time parametric imaging (At-PI) findings on day 22. Hepatic segment 8 is highlighted in yellow indicating an early enhancement time. Other areas of the hepatic parenchyma are in blue, indicating a late enhancement time.

The results of At-PI on admission showed that the entire right lobe of the liver was highlighted in yellow due to the portal vein obstruction by the thrombus, revealing that the liver parenchyma was perfused by the hepatic artery (Fig. 6). The abatement of the patient’s fever and an improvement in sepsis began on hospital day 8 and the blood culture was then negative on day 9. With the exception of one thrombus in the P8 branch of the portal vein, virtually complete dissolution of the thrombi in the right main branch of the portal vein and in the superior mesenteric vein was observed by abdominal B-mode US on day 22 (Figs. 7 and 8). At-PI performed on this day displayed a yellow color over segment 8 of the liver parenchyma indicative of early contrast arrival times, while the other areas of the liver parenchyma were displayed in blue, suggesting late arrival times (Fig. 9). In essence, segment 8 continued to receive hepatic arterial perfusion due to the undissolved thrombus although portal venous perfusion had been restored in the rest of the parenchyma. An oral administration of 2 mg warfarin was started upon discharge. The hepatic parenchyma in segment 8 remained yellow in At-PI images at 6 months after discharge, as was observed on hospital day 22.

## Discussion

At-PI is an ultrasound imaging analysis tool that was introduced into the Toshiba Aplio XG ultrasound system (Toshiba Medical Systems Co.) in October 2010 and which traces and color codes temporal changes in contrast-enhanced US images. Using At-PI, we have previously investigated the effect of the hepatic portal vein and the hepatic artery on hepatic parenchymal enhancement and also reported its use in the clinical assessment of type C chronic liver disease (5) and alcoholic liver disease (6). In the present study, At-PI was performed to elucidate the effect of a portal thrombus on hepatic parenchymal enhancement and to demonstrate the changes in the contrast subsequent to the treatment.

In the present study, the best use of At-PI technology in the evaluation of hepatic parenchymal perfusion was considered to be in the comparison of the arrival times of the Sonazoid to the liver and kidney. While the liver receives its blood supply from the hepatic artery and the portal vein, the kidney is supplied exclusively by the arteries. A comparison of the contrast starting times between the liver and kidney parenchyma provides an insight into the balance of the hepatic blood flow. A shortened time difference between the hepatic and renal enhancement may indicate a change in the hepatic blood flow from portal vein dominance to hepatic artery dominance, enabling an objective evaluation of the hepatic hemodynamic balance between the portal vein and hepatic artery.

In line with the objectives and considerations mentioned above, the present study revealed that, due to a blocked right portal vein branch at admission, the right hepatic lobe was perfused by the hepatic artery, as indicated by a yellow color representing the early contrast enhancement. Subsequent to starting the anticoagulant therapy, another At-PI performed on hospital day 22 revealed that the liver parenchyma, with the exception of segment 8, was in blue, indicating that the portal vein had resumed its role of being the major blood supply to the liver.

The blood flow in the liver, particularly the intrahepatic microcirculation, requires consideration when studying the changes in hepatic parenchymal perfusion. Sonazoid injected via the cubital vein reaches the liver via the arterial blood flow. In healthy individuals, the hepatic artery is considered to be the blood vessel that feeds the biliary system. Arterial blood flow nourishes the large and small bile ducts while traveling through the liver toward the periphery. A peribiliary capillary plexus is formed around the bile ducts and a number of the capillaries merge into the terminal portal venules or sinusoids. There is, however, a population of branches that directly merge into the sinusoids without passing through the peribiliary capillary plexus or nourishing the portal vein by extending to the wall (7–9).

The ratio of blood inflow via the hepatic portal vein and the hepatic artery is 7–8:2–3 and therefore the portal vein is the main blood supply to the liver in healthy individuals (1). This is presumably due to the blood flowing through the portal vein containing nutrients from the stomach and intestine, making the portal vein the nutrient vessel for the liver. A previous study, which examined the pressure difference between the two vascular systems by micropuncturing the hepatic microvasculature under a biomicroscope, reported that blood pressure at the distal end branches of the hepatic artery was 6–8 times higher than that at the portal vein branches (300–400 vs. 50 mm H_2_O, respectively) (10). The precapillary sphincter plays a significant role in enabling the portal vein to carry a large amount of blood into the sinusoids despite its low blood pressure. The distal end branches of the hepatic artery and the peribiliary capillary plexus around the bile ducts exist in the form of capillaries. Each capillary is encircled by a precapillary sphincter which adjusts the blood flow from the arterioles into the capillary (11). By controlling the amount of blood entering from the high-pressure arterial system, the precapillary sphincter makes it easier for the low-pressure portal vein system to pump blood into the sinusoids.

The present study revealed that when the portal vein blood flow is obstructed by a thrombus, the hepatic artery takes over parenchymal perfusion of the affected liver segments. It is likely that obstruction of the portal vein blood flow triggers a chain of signal transduction that allows the precapillary sphincter to increase the arterial blood flow to compensate for the reduced blood flow to the liver. When thrombus dissolution improved portal vein blood flow to the liver parenchyma in our patient, hepatic perfusion restored portal vein dominance, presumably through a readjustment of the arterial blood flow by the precapillary sphincter.

In conclusion, the present study of acute portal vein thrombosis demonstrates how a portal thrombus affects hepatic parenchymal enhancement and how anticoagulant treatment changes the pattern of contrast enhancement. At-PI also enabled the easy visualization of the contrast changes and provided a deeper understanding of the pathology. Even 6 months subsequent to discharge, the hepatic parenchymal perfusion in segment 8 continued to show a yellow color reflecting the arterial blood supply. If hepatic parenchymal perfusion remains arterialized subsequent to the treatment of the portal vein thrombosis, as in our patient, then it may be necessary to administer a more powerful anticoagulant, such as urokinase, and to start thrombolytic therapy as an additional intervention. Further study is warranted to investigate the utility of At-PI in the treatment of arterialization and to accumulate a higher number of cases.

## Figures and Tables

**Table I.** Hematological findings on admission.

Diagnostic blood tests	Results
White blood cells	16,900 cells/*μ*l
Red blood cells	460×10^4^ cells/*μ*l
Hemoglobin	14.6 g/dl
Platelets	3.7/*μ*l
Basophils	0%
Eosinophils	1.3%
Lymphocytes	27.8%
Monocytes	5.2%
Neutrophils	65.6%
Prothrombin time	82%
Activated partial thromboplastin time	35.2 sec
Total protein	5.2 g/dl
Albumin	1.9 g/dl
Thymol turbidity test	3.2 SH-U
Zinc turbidity test	9.5 K-U
Total bilirubin	4.4 mg/dl
Direct bilirubin	2.8 mg/dl
Aspartate aminotransferase	65 IU/l
Alanine aminotransferase	44 IU/l
Lactate dehydrogenase	219 IU/l
Alkaline phosphatase	746 IU/l
γ-glutamyltranspeptidase	176 IU/l
Choline esterase	78 IU/l
Amylase	31 IU/l
Blood urea nitrogen	34 mg/dl
Creatinine	1.25 mg/dl
Na	131 mM
K	3.2 mM
Cl	109 mM
C-reactive protein	21.0 mg/dl
Fibrin degradation product	18.7 mg/dl
D-dimer	10.4 mg/dl
Hepatitis C virus antibody	-
Hepatitis B surface antigen	-
Carcinoembryonic antigen	3.9 ng/ml
Carbohydrate antigen 19-9	9.6 U/ml

## Abbreviations:

At-PI: arrival time parametric imaging;
US: ultrasonography;
CRP: C-reactive protein;
CT: computed tomography;
ROI: region of interest

## References

[1] Kleber G, Steudel N, Behrmann C (1999). Hepatic arterial flow volume and reserve in patients with cirrhosis: use of intra-arterial Doppler and adenosine infusion. Gastroenterology.

[2] Rocheleau B, Ethier C, Houle R, Huet PM, Bilodeau M (1999). Hepatic artery buffer response following left portal vein ligation: its role in liver tissue homeostasis. Am J Physiol.

[3] Leen E, Goldberg JA, Anderson JR (1993). Hepatic perfusion changes in patients with liver metastases: comparison with those patients with cirrhosis. Gut.

[4] Lautt WW (1985). Mechanism and role of intrinsic regulation of hepatic arterial blood flow: hepatic arterial buffer response. Am J Physiol.

[5] Wakui N, Takayama R, Kanekawa T (2012). Usefulness of arrival time parametric imaging in evaluating the degree of liver disease progression in chronic hepatitis C infection. J Ultrasound Med.

[6] Wakui N, Takayama R, Mimura T (2011). Drinking status of heavy drinkers detected by arrival time parametric imaging using sonazoid-enhanced ultrasonography: study of two cases. Case Rep Gastroenterol.

[7] Rappaport AM, Black RG, Locas CC, Ridout JH, Best CH (1966). Normal and pathologic microcirculation of the living mammalian liver. Rev Int Hepatol.

[8] Rappaport AM (1980). Hepatic blood flow: morphologic aspects and physiologic regulation. Int Rev Physiol.

[9] Ekataksin W, Kaneda K (1999). Liver microvascular architecture: an insight into the pathophysiology of portal hypertension. Semin Liver Dis.

[10] Nakata K, Leong GF, Brauer RW (1960). Direct measurement of blood pressures in minute vessels of the liver. Am J Physiol.

[11] Rhodin JA (1967). The ultrastructure of mammalian arterioles and precapillary sphincters. J Ultrastruct Res.

